# Costing recommended (healthy) and current (unhealthy) diets in urban and inner regional areas of Australia using remote price collection methods

**DOI:** 10.1017/S1368980021004006

**Published:** 2022-03

**Authors:** Christina Zorbas, Ruby Brooks, Rebecca Bennett, Amanda Lee, Josephine Marshall, Shaan Naughton, Meron Lewis, Anna Peeters, Kathryn Backholer

**Affiliations:** 1 Deakin University, Global Obesity Centre, Institute for Health Transformation, 1 Gheringhap Street, Geelong, VIC, Australia; 2 The University of Queensland, The School of Public Health, Brisbane, QLD, Australia

**Keywords:** Food price, Food costs, Diet affordability, Price monitoring

## Abstract

**Objective:**

To compare the cost and affordability of two fortnightly diets (representing the national guidelines and current consumption) across areas containing Australia’s major supermarkets.

**Design:**

The Healthy Diets Australian Standardised Affordability and Pricing protocol was used.

**Setting:**

Price data were collected online and via phone calls in fifty-one urban and inner regional locations across Australia.

**Participants:**

Not applicable.

**Results:**

Healthy diets were consistently less expensive than current (unhealthy) diets. Nonetheless, healthy diets would cost 25–26 % of the disposable income for low-income households and 30–31 % of the poverty line. Differences in gross incomes (the most available income metric which overrepresents disposable income) drove national variations in diet affordability (from 14 % of the median gross household incomes in the Australian Capital Territory and Northern Territory to 25 % of the median gross household income in Tasmania).

**Conclusions:**

In Australian cities and regional areas with major supermarkets, access to affordable diets remains problematic for families receiving low incomes. These findings are likely to be exacerbated in outer regional and remote areas (not included in this study). To make healthy diets economically appealing, policies that reduce the (absolute and relative) costs of healthy diets and increase the incomes of Australians living in poverty are required.

Dietary risks are among the leading risk factors for premature morbidity and mortality globally^(1,2)^. These risk factors comprise diets high in discretionary foods and beverages (which contain excessive amounts of added sugars, saturated fats, salt and/or alcohol) and diets low in whole grains, fruits, vegetables, legumes, nuts and seeds and dairy (i.e. healthy or recommended foods and beverages)^(2,3)^. Despite international health agendas having long identified the need to create healthy food environments to shift population diets towards healthier patterns and reduce the global burden of disease^(4,5)^, high-level actions have been inadequate^(6,7)^. There is widespread recognition by public health experts, and members of the public, that the cost and affordability of foods and beverages continue to pose major obstacles to the purchase and consumption of healthy foods and diets over unhealthy foods and diets^(8–10)^.

The 2020 United Nations Report on *The State of Food Security and Nutrition in the World – Transforming Food Systems for Affordable and Healthy Diets* clearly conveys how food and beverage costs and their affordability are key determinants of malnutrition globally – including both undernutrition and obesity^(8,11)^. The findings from the report estimate that in 2017, healthy diets (which include eating diverse and culturally specific foods from multiple food groups) were priced 60 % higher than diets that only met the essential nutrient requirements (based on a few food items) across 170 low-, middle- and high-income countries. Moreover, healthy diets were estimated to be unaffordable for 3 billion people. It should be noted that the affordability of foods, beverages and diets is a function of both their cost and a household’s income. With recent global events, such as the COVID-19 pandemic and climate change (e.g. the 2019–2020 Australian bushfires), severely impacting employment and food systems, we may see unfavourable impacts on people’s incomes and the price of foods and beverages^(8)^. Combined, these factors highlight the urgent need for ongoing monitoring of the price and affordability of healthy and current (unhealthy) diets.

While a number of studies have assessed the cost and affordability of healthy and unhealthy foods, beverages and diets internationally and within Australia^(12–15)^, surveys have been infrequently conducted and have typically been limited to defined geographical regions. In part, food and beverage price monitoring has been limited by the resource-intensive nature of data collection, which traditionally requires researchers to physically travel to different retail outlets to collect prices in-store. To reduce the resource intensiveness of food and beverage price data collection and increase the scale of data collection across geographical regions, there is potential to capitalise on the increasing availability of online food and beverage price data^(12,16)^.

The use of a wide variety of tools and methods to measure the cost or affordability of healthy and unhealthy foods, beverages and diets has also limited the comparability of analyses across different regions^(17)^. In light of this, the Healthy Diets Australian Standardised Affordability and Pricing (HD-ASAP) protocol^(18)^ was developed to provide a standardised and optimal approach to assessing the cost, cost-differential and affordability of the current (unhealthy) Australian diet^(19)^ and a healthy (recommended) diet^(3)^. Using the HD-ASAP protocol, we recently compared estimates of the cost and affordability of healthy and current (unhealthy) diets using data collected in-store with data collected online and by phone calls in Victoria, Australia^(12)^. This pilot study illustrated that, in major cities and inner regional areas where major supermarkets are present, collecting data online and by phone calls can be both reliable and significantly less resource-intensive compared with traditional in-store methods. Low-resource food and beverage price monitoring have been recognised by multiple United Nations organisations and international experts as pivotal to informing appropriate regulatory measures that promote and protect the affordability of healthy diets, over and above unhealthy options, globally^(8,16,20)^.

To date few studies have estimated the cost and affordability of healthy and current (unhealthy) diets across an entire nation owing to the difficulty and resources required to collect such data in-store across geographically disperse areas. Whilst optimal methods to cost diets continue to evolve within Australia, one the most geographically disperse and diverse regions in the world, they have never been applied to more than eighteen local areas in one or two of Australia’s eight States or Territories (i.e. small scale piloting)^(21)^. We aimed to address this gap by upscaling our reliable lower-resource price monitoring methods to determine whether the cost, cost differential and affordability of healthy (recommended) and current (unhealthy) diets varied across areas where major supermarkets are present in Australia. It is important to note that due to the absence of major supermarkets, outer regional, remote and very remote areas were not included in the current analysis.

## Methods

### Study design

A cross-sectional study was conducted using publicly available food and beverage price data collected online if available (supermarket, alcohol and some fast-food chains) and by phone calls from all other outlets (fast-food chains, independent bakeries, fish and chip shops and convenience stores). Food and beverage price data were collected over a 2-week period in May 2019. This period did not include any festive events, such as Easter, that may have affected the results.

### The healthy diets Australian standardised affordability and pricing protocol

The current study was guided by the HD-ASAP protocol for measuring the cost and affordability of healthy (recommended) and current (unhealthy) diets in Australia, which has been described in detail elsewhere^(18)^. The healthy diet reflects the recommendations of the Australian Dietary Guidelines^(3)^ (and comprises forty-three representative foods and beverages across seven food groups). The current (unhealthy) diet reflects mean dietary intakes reported for selected age/gender groups in the National Nutrition and Physical Activity Survey of the Australian Health Survey 2011–2013^(19)^. The current diet comprises the same core items as the healthy diet in different amounts plus thirty-two representative unhealthy foods and beverages in amounts that exceed dietary recommendations (see Table 1). For example, the diets assume that a household currently consumes 0·9 kg of bananas per fortnight compared with 5·5 kg required to meet recommended fruit intakes and 0·6 kg of table sugar compared with no added sugar as per dietary recommendations (specific diet details have been published elsewhere^(14)^).

**Table 1:** Foods included in the healthy diets Australian standardised affordability and pricing (HD-ASAP) protocol*

Foods and beverages included in the healthy (recommended) diet	Foods and beverages included in the current (unhealthy) diet, in addition to foods included in the healthy diet in reduced amounts†
Water (bottled) Fruit: apples, bananas, oranges Vegetables: potatoes, broccoli, white cabbage, iceberg lettuce, onion, carrot, pumpkin, tomatoes, sweet corn (canned), four bean mix (canned), diced tomatoes (canned), baked beans (canned), frozen mixed vegetables, frozen peas, salad vegetables in sandwich Grain (cereals): wholegrain cereal biscuits (Weet-bix™), rolled oats, cornflakes, whole meal bread, white bread, white rice, white pasta, dry water cracker biscuits, bread in sandwich Meats and alternatives: beef mince, lamb chops, beef steak, cooked chicken, tuna (canned), eggs, peanuts (unsalted), chicken in sandwich Dairy and alternatives: cheddar cheese (full fat, reduced fat), milk (full fat, reduced fat), yoghurt (full fat plain, reduced fat flavoured) Oils: sunflower, olive, canola (margarine)	Beverages: artificially sweetened soft drink, sugar sweetened soft drink, orange juice Processed cereals, snacks and desserts: sweet muffins, sweet biscuits, savoury biscuits, confectionary, chocolate, potato crisps, muesli bars, mixed nuts (salted), ice cream, fruit salad (canned in juice) Processed meats: beef sausages, ham Spreads, sauces, condiments and ingredients: butter, tomato sauce, salad dressing, white sugar Convenience meals: frozen lasagne, chicken soup (canned), frozen fish fillets (crumbed), instant noodles, meat and vegetable casserole (canned) Fast-food: pizza, meat pie, hamburger, potato chips/fries Alcohol: beer (full strength), white wine (sparkling), red wine, whisky

* Table sourced from Zorbas *et al.* ^(12)^ (adapted from Lee *et al*.^(18)^).

† Unhealthy diets reflect current food and beverage consumption levels for a reference family across the Australian population according to the 2011–2013 Australian Health Survey^(19)^.

Both diets are adjusted to reflect fortnightly consumption for a reference household consisting of four people: an adult male 31–50 years old, an adult female 31–50 years old, a 14-year-old boy, and an 8-year-old girl. For this family, 35 % of the energy provided by the current diet is derived from ‘discretionary’ foods and beverages, that is, those that are not required for health and are high in added sugars, saturated fats salt and/or alcohol^(19)^. Compared with the current diet, the healthy diet is more environmentally sustainable^(3)^, requiring less water, supporting greater biodiversity and having a lower carbon footprint (on average 25 % less Green House Gas emissions)^(3,22)^. The total energy content of both household diets are similar; 33610 kJ/d for the healthy diet and 33860 kJ/d for the current (unhealthy) diet^(18)^.

To cost the healthy and current (unhealthy) diets, the prices of a predetermined list of foods and beverages (Table 1; full protocol also includes product brands and sizes that are commonly purchased by Australians, including fast-food and alcohol) are collected from specific food retailers in a selected area^(18)^ (described in further detail below).

### Sample

We used simple stratified random sampling to select a sample of areas where food and beverage stores and prices were collected. The Statistical Area 2 (SA2) geographical unit was used for sampling. These units are Australian Bureau of Statistics – defined areas that represent communities (between 3000 and 25 000 people) that interact socially and economically^(23)^. All SA2s across the eight Australian States and Territories were eligible for inclusion except for SA2s where major supermarkets do not operate. This resulted in the exclusion of eighteen non-spatial special purpose SA2s and SA2s for the ‘Other Territories’ (Jervis Bay, Cocos (Keeling) Island, Christmas Island and Norfolk Island). All remaining SA2s were classified twofold: according to relative socio-economic disadvantage and remoteness. SA2s were classified into quintiles of relative disadvantage at the State/Territory level based on the Index of Relative Socio-economic Disadvantage (IRSD; Q1: most disadvantaged, Q5: least disadvantaged^(24)^). By remoteness, SA2s were classified using the Accessibility/Remoteness Index of Australia (ARIA^+^)^(25)^. The two major supermarket chains of interest were only found in ‘Major cities’ (ARIA^+^ 1) or ‘Inner regional areas’ (ARIA^+^ 2), thus our study was restricted to these regions and could provide no information about food prices in outer regional, remote or very remote areas. If a state or territory did not include an IRSD or ARIA^+^ stratum, the stratum was excluded from the study.

To systematically sample SA2s, each SA2 was assigned a consecutive number, within each IRSD quintile and ARIA^+^ category, for each Australian State or Territory. We aimed to sample one SA2 to align with each IRSD and ARIA^+^ stratum using a random number generator. The inclusion of selected SA2s was confirmed if it contained the two major Australian supermarket chains and their alcohol chains. When a selected SA2 did not meet this requirement (ARIA^+^ 1/major cities = 200 SA2s excluded, 15 %; ARIA^+^ 2/inner regional areas = 103 SA2s excluded, 21 %), a new SA2 was randomly selected.

Our sample of retailers included the two major supermarkets and their alcohol chains (which possess approximately two-thirds of the grocery market share and are most likely to represent food and beverage prices in Australia^(26)^). Non-supermarket retailers that sell commonly consumed fast-food items^(27)^ (McDonald’s, Domino’s, independent bakeries, fish and chip shops and convenience stores or gas service stations such as 7 Eleven for a pre-prepared chicken sandwich) were also included and sampled using Google Maps, with the most central retailers selected. These selected retailers were all required to be within seven kilometres of the centre of the SA2 (as per the original HD-ASAP protocol^(18)^).

### Data collection

Prices for the predetermined list of foods and beverages were collected online from two supermarkets, two alcohol stores and one of each of the fast-food chains (as specified). The researcher’s geolocation was set to the selected SA2 (on the online platform) before price collection began. Foods and beverages were located using the website’s search function. When an item was not listed online, prices for the cheapest, similarly sized, branded item were recorded (as per HD-ASAP approach). Prices for included foods from independent bakeries (beef pie), fish and chip shops (small/minimum chips) and convenience stores (chicken sandwich), as well as roast chicken from a supermarket, were collected by phone call.

In Tasmania, data collection was affected by the absence of one of the alcohol retail chains; therefore, alcohol prices from a single retail chain were used when estimating diet costs for Tasmania. One Tasmanian SA2 (in the second SEIFA quintile) was excluded due to a data collection error for one major supermarket chain and non-supermarket items.

### Data analyses

The mean *cost*, *cost differential* and *affordability* of the healthy and current (unhealthy) diets were calculated for each State and Territory. A standardised HD-ASAP template was used to convert the price per unit for each food and beverage item to the price per edible gram or millilitre ($AUD). The prices of supermarket and alcohol items were averaged across the two stores in each SA2 and supplemented with prices collected from individual fast-food stores. For each food or beverage item, this was then multiplied by the amount recommended (healthy) or currently consumed (unhealthy) by the reference household for a fortnight. These costs were then summed to determine the *diet costs*. No consistent trends were observed for differences in diet costs across IRSD categories or major cities/inner regional ARIA^+^ areas (see online supplemental Table S1). As such, the results were aggregated at the State level.

The absolute *cost differential of the healthy and current (unhealthy) diet* for each State and Territory was calculated in dollars (by subtracting the mean cost of the healthy diet from that of the unhealthy diet) and as a relative percentage difference (mean differential in dollars divided by the mean cost of the unhealthy diet). We additionally calculated and reported the mean fortnightly costs of key food groups within each diet, for the reference household. These included fruits; vegetables and legumes; grains and cereals; meats, nuts, seeds and eggs; milk, yoghurt and cheese; alcoholic beverages; take-away foods and soft drinks. The State-level estimates of the cost of a healthy diet, a current (unhealthy) diet and each food group are represented as means and standard deviations across all SA2s in each State or Territory. Mean costs were compared between States and/or Territories using Mann–Whitney *U* tests (*α* level of 0·05) in Stata 16.

The mean *affordability* of the healthy and current (unhealthy) diets in each State and Territory was first assessed against the national poverty line. In 2017–2018, this was $960/week for a couple with two children (not taking into account housing costs)^(28)^. Affordability was also assessed against a national indicative low disposable household income that estimates a minimum wage-based household disposable income calculated in line with the HD-ASAP protocol (see online supplemental Table S2)^(18)^. For both denominators, a diet affordability threshold of 30 % was used (i.e. diet costs should not exceed 30 % of the minimum incomes available to Australians experiencing economic hardship)^(18)^.

Finally, to enable area-level comparisons, affordability was assessed against the median total gross household income (which is the only readily available metric) for each State and Territory (Australian Bureau of Statistics, 2016 Census^(29)^). Total gross household income was adjusted by the wage price index as per the HD-ASAP protocol^(18)^.

## Results

A total of 51 SA2s were sampled in the current study (see online supplemental Tables S3 and S4). This varied across States and Territories, from two eligible SA2s in the Australian Capital Territory (ACT) (where there were no SA2s classified in the IRSD Q1–3 or inner regional strata) to 10 SA2s in Victoria (one per IRSD quintile across major cities and inner regional ARIA^+^ areas). Each SEIFA quintile contained 8–12 SA2s and major cities/inner regional areas each contained 26 SA2s. Food and beverage price data were collected from a total of 455 retail stores (*n* 102 supermarket, *n* 98 alcohol, *n* 51 McDonald’s, *n* 51 Domino’s, *n* 51 bakeries, *n* 51 fish and chips and *n* 51 convenience stores).

### Diet costs and cost differentials

**Fig. 1:**
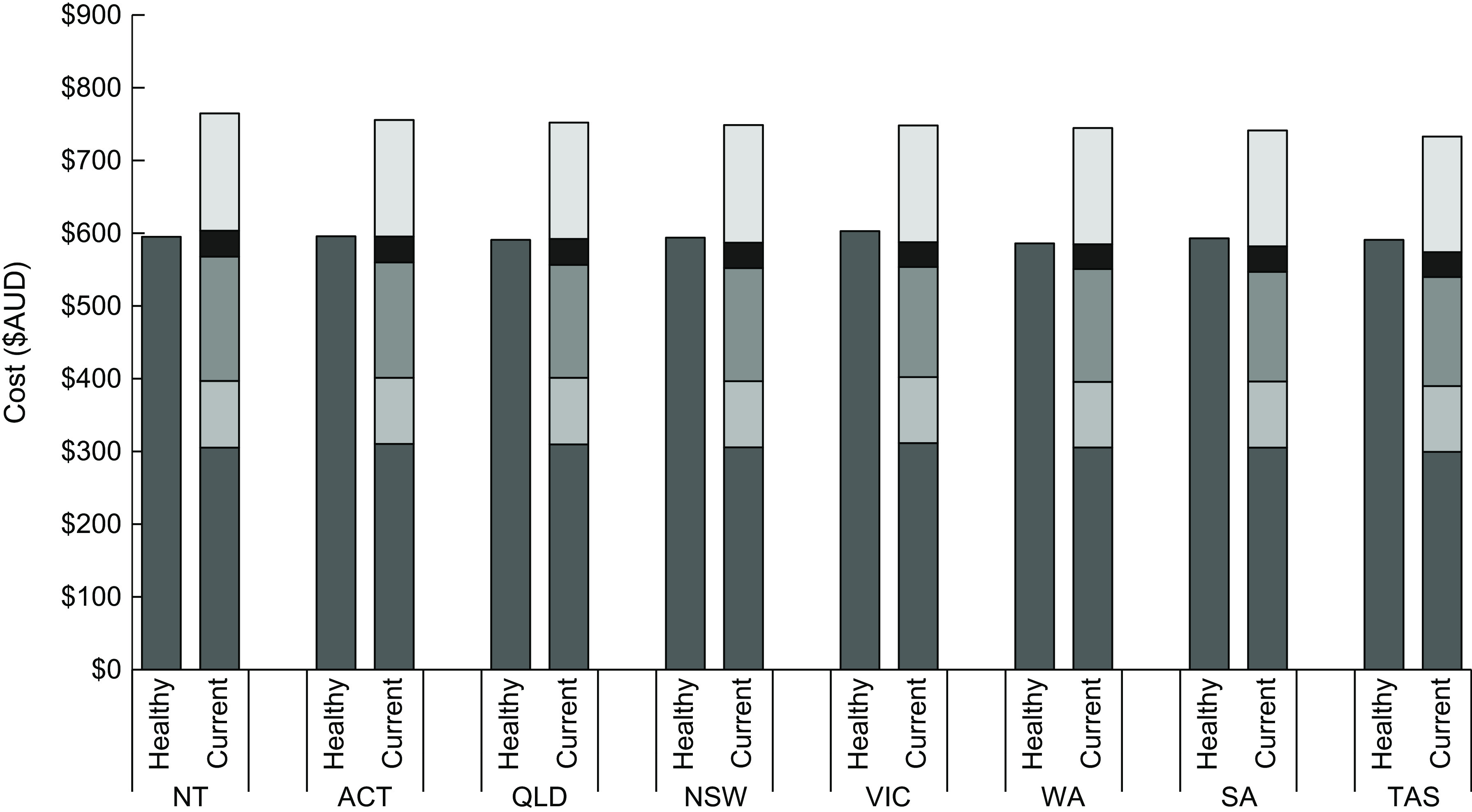
Mean fortnightly cost of healthy and current (unhealthy) diets, by State and Territory, May 2019. ACT: Australian Capital Territory, Qld: Queensland, NSW: New South Wales, Vic: Victoria, WA: Western Australia, SA: South Australia, Tas: Tasmania, NT: Northern Territory. 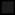, Healthy food groups; 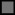, Discretionary, Alcohol; 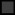, Discretionary, Take-away; 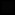, Discretionary, Soft drinks; 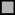, Discretionary, Other

For all States and Territories, the mean fortnightly cost of the current (unhealthy) diet for the reference household of four people was more expensive than that of the healthy diet (Table 2). There was some variation in the costs of the diets (healthy and unhealthy) within and between States and Territories (Fig. 1; Table 2). The absolute and relative cost differentials (between a healthy and unhealthy diet) were lowest for the Northern Territory (NT) ($139·38 per fortnight; 19 %) and highest for Queensland ($159·89 per fortnight; 21 %). The healthy diet was $16·78 (3 %) per fortnight more expensive in Victoria, where it was most expensive ($602·72 per fortnight), compared with Western Australia, where it was cheapest ($585·94 per fortnight). The current (unhealthy) diet was $33·32 (5 %) more expensive per fortnight in the NT, where it was most expensive ($764·68), compared with Tasmania, where it was cheapest ($732·85).

**Table 2:** Mean fortnightly cost and affordability of healthy and current (unhealthy) diets consumed by a reference household of two adults and two children, by state and territory, May 2019

	Tas (*n* 4)		SA (*n* 5)		WA (*n* 9)		Vic (*n* 10)		NSW (*n* 9)		Qld (*n* 9)		ACT (*n* 2)		NT (*n* 3)	
	Mean	sd	Mean	sd	Mean	sd	Mean	sd	Mean	sd	Mean	sd	Mean	sd	Mean	sd
Cost, $AUD																
Healthy	590·75	1·17	593·05	5·13	585·94	6·09	602·72*	1·76	593·85	2·60	590·74	3·57	595·70	1·26	595·14	3·39
Current	732·85	12·88	741·26	3·92	744·55	12·82	748·15	6·24	748·82	5·12	752·07	16·25	755·74	6·28	764·68	3·92
Cost differential																
Mean %	19·39		19·99		21·30		19·44		20·70		21·45		21·18		22·17	
Affordability†, national poverty line																
Healthy																
%	30·77		30·89		30·52		31·39		30·93		30·77		31·03		31·00	
Current																
%	38·17		38·61		38·78		38·97		39·00		38·09		39·36		39·83	
Affordability, indicative low disposable household income																
Healthy																
%	25·40		25·50		25·19		25·91		25·53		25·40		25·61		25·59	
Current																
%	31·51		31·87		32·01		32·17		32·19		32·33		32·49		32·88	
Affordability, median gross household income																
Healthy																
%	25·09		23·08		17·58		19·75		18·73		19·77		13·57		14·21	
Current																
%	31·12		28·84		22·34		24·52		23·62		25·17		17·22		18·26	

* *P* < 0 05 (Mann–Whitney *U* tests; Tasmania used as reference group due to having the cheapest healthy and current diet costs).

† Affordability estimates calculated using the State and Territory mean diet costs.

### Food group costs

Table 3 outlines the cost of each food group, within the healthy and current (unhealthy) diets, across each State and Territory. Within the healthy diet food groups, the cost of grains and cereals and meats, nuts, seeds and eggs were similar across all States and Territories. In comparison, variations of most to least expensive were in the cost of milk, yoghurt and cheese (16 % more expensive in Western Australia compared with New South Wales), fruit ($11–12 per fortnight or 15–16 % more expensive in the NT and ACT compared with Queensland) and vegetables and legumes (9 % more expensive in Victoria compared with South Australia). The costs of alcoholic beverages and soft drinks were relatively stable across each State and Territory. Take-away foods were most expensive in the NT and cheapest in Tasmania (14 % cost difference).

**Table 3:** Mean fortnightly costs ($AUD) of key food and beverage groups in current (unhealthy) and healthy amounts consumed by a reference household of two adults and two children, by state and territory, May 2019

	Tas (*n* 4)		SA (*n* 5)		WA (*n* 9)		Vic (*n* 10)		NSW (*n* 9)		Qld (*n* 9)		ACT (*n* 2)		NT (*n* 3)	
	Mean	sd	Mean	sd	Mean	sd	Mean	sd	Mean	sd	Mean	sd	Mean	sd	Mean	sd
Fruit (current)	53·65	1·14	51·08	1·72	52·01*	1·40	52·40*	2·15	50·74*	1·17	50·13*	1·66	55·54	0·00	51·81	1·31
Fruit (healthy)	85·95	0·00	79·00*	3·13	81·28*	2·21	80·38*	0·09	74·45*	3·55	74·39*	2·56	86·25	0·00	85·61	0·00
Vegetables and legumes (current)	42·21	1·36	40·35*	0·31	43·04*	0·88	44·66*	0·89	44·28*	0·61	42·79	0·77	43·73	1·81	41·84	0·16
Vegetables and legumes (healthy)	100·88	0·18	96·10*	1·93	101·07*	3·48	104·55*	1·73	103·88*	1·30	101·22	1·93	102·11	0·85	97·99	0·32
Grains and cereals (current)	40·30	1·04	42·75*	0·44	43·33*	0·75	43·30*	0·83	43·01*	0·66	42·92*	0·52	43·57	1·79	43·64	0·39
Grains and cereals (healthy)	97·83	1·21	101·78*	0·00	101·83*	0·16	101·16*	0·00	101·59*	0·38	101·33*	0·61	101·47	0·44	102·46	0·87
Meats, nuts, seeds and eggs (current)	100·79	1·32	102·61*	0·45	103·18*	0·86	103·24*	0·83	103·32*	0·84	104·11*	1·91	101·91	3·27	104·63	0·87
Meats, nuts, seeds and eggs (healthy)	190·34	1·41	192·47*	0·34	192·65*	1·30	192·37*	0·00	192·70*	0·63	193·23*	2·17	190·72	1·80	195·18	1·87
Milk, yoghurt and cheese (current)	36·74	1·21	41·89*	0·24	39·70*	0·24	42·24*	0·20	42·22*	0·44	38·61	0·25	38·73	1·30	39·29	0·43
Milk, yoghurt and cheese (healthy)	89·18	0·68	96·48*	0·21	84·09*	0·43	97·70*	0·57	94·12*	0·47	92·90*	0·20	87·71	1·84	89·12	1·60
Discretionary, alcoholic beverages (current)	90·55	0·00	91·07*	0·00	90·26*	0·67	90·55	0·00	91·07*	0·00	91·75*	0·25	91·07	0·00	91·73	0·00
Discretionary, take-away foods (current)	149·95	10·00	150·57	3·94	154·99	10·93	151·44	6·69	155·58	3·79	159·18	13·48	158·66	12·45	170·86	0·63
Discretionary, soft drinks (current)	34·09	0·00	35·11*	0·25	34·09	0·00	34·09	0·00	34·69*	0·72	35·70*	0·28	35·37	0·59	35·60	0·81
Discretionary, other (current)	158·89	1·14	159·32	1·00	159·82	1·13	160·54*	0·17	161·98*	3·97	159·84	1·01	160·39	0·08	161·25	0·66

* *P* < 0 05.

Mann–Whitney *U* tests; Tasmania used as reference group due to having the least expensive healthy and current diet costs.

### Diet affordability

When assessed against the national poverty line (same across all States and Territories), both the healthy and current (unhealthy) diets were considered unaffordable across all major cities and inner regional areas (costing 31 % and 38–40 % of the value of the poverty line, respectively) (Table 2). Against the indicative low disposable income (also the same across the nation but higher than the poverty line), the healthy diet was considered affordable (25–26 % of income), but the current (unhealthy) diet was not (31–33 %).

When assessed against the median gross household income (noting that gross income overrepresents the affordability of diets compared with disposable income) that varied between States and Territories, there was greater variation in the affordability of the diets as a percentage of income (Table 2). While the healthy diet was considered affordable for all major cities and regional areas across States and Territories, it made up only 14 % of gross income in both the ACT and the NT (noting that state-level gross incomes can overrepresent disposable incomes for lower income households, particularly in remote areas) compared with 25 % in Tasmania. Similarly, the current (unhealthy) diet cost only 17–18 % of the gross incomes in both the ACT and the NT compared with 31 % in Tasmania.

## Discussion

In 2019, an Australian household would find healthy diets to be less expensive and more affordable than current (unhealthy) diets in all major cities and inner regional areas sampled. The higher cost of the current diet (compared to the healthy diet) is due to the additional costs incurred from the consumption of excessive amounts of unhealthy foods and beverages. This occurs despite the healthy and current diets being similar in energy content; per reference household, the current diet provides 33 860 kJ/d and the recommended diet provides 3600 kJ/d^(18)^. Similar results have been observed in several studies in Australia^(12,21,30)^ and New Zealand^(31)^.

This evidence is contrary to existing perceptions and literature that suggest healthy diets are more expensive than current (unhealthy) diets by on average by $US 1·50/person/d^(10,32)^. In comparison, standardisation of our estimates indicates that healthy diets are more expensive than current (unhealthy) diets by approximately $US 2/person/d in Australia. One explanation for this discrepancy reflects the differences in methodological approaches, particularly given the wide range of selected food and beverage items that make up the different diets across studies^(33)^. Notably, unlike many other methods, the HD-ASAP protocol includes alcohol and high intakes of fast foods when estimating current (unhealthy) diet costs, but not in the healthy diets as these items are not included in Australia’s national dietary guidelines^(3)^. This adds additional costs to the current (unhealthy) diet (i.e. alcoholic beverages comprising 12 % of the current food budget in Australia^(21)^), making the healthy diet relatively more affordable than when they are not included.

Importantly, the relative affordability of healthy compared with current (unhealthy) diets in Australia is likely, in part, attributed to the 10 % Goods and Services Tax (GST) exemption for all basic, healthy foods^(34)^. Furthermore, whilst we found that healthy diets were more affordable compared with unhealthy (current) diets across Australia, few methodological approaches consider the time costs associated with preparing healthy foods^(35)^ or how price promotions encourage consumers to make purchasing decisions (often for unhealthy options) on a food-by-food rather than whole-of-diet basis^(10,36)^. These factors are also likely to contribute to current perceptions around the relative affordability of current (unhealthy) over healthy diets and interplay with many other factors to influence food consumption practices (e.g. food access, income, etc.)^(10,21)^.

We must note that our results do not reflect State- or Territory-wide diet cost or affordability estimates as we could not sample outer regional, remote or very remote areas where there are no major supermarkets. Nonetheless, we demonstrated that both healthy and current (unhealthy) diets would be unaffordable across all Australian jurisdictions for households living on the poverty line. Interstate affordability differences were primarily observed when assessed against median gross household incomes. That is, a healthy diet would be most affordable in major cities and inner regional areas in the ACT and NT (where median gross household incomes are highest) and least affordable in Tasmania. These findings highlight the need to ensure all households receive adequate incomes that provide the opportunity to afford and consume a healthy diet.

### Diet cost variability in Australia

When assessing the average fortnightly cost of a healthy diet in major cities and inner-regional areas, we found that it differed between States and Territories, with a maximum difference of $16·78 ($585·94 in Western Australia to $602·72 in Victoria, equating to an additional $436 per annum for a family living in a major city or inner regional area in Victoria). These differences were largely driven by milk, yoghurt and cheese prices. Across 1 year, milk, yoghurt and cheese in the healthy diet would be approximately $354 more expensive for a family living in a major city or inner-regional area in Victoria compared with Western Australia. Between States and Territories, the maximum difference in the average fortnightly cost of the current (unhealthy) diet was $33·32, with differences predominantly driven by variations in the price of take-away foods. The cost of take-away foods would be approximately $540 more expensive, per annum, for a family in inner regional areas (no other ARIA^+^ areas sampled) of the NT compared with Tasmania.

### Diet affordability in Australia

When assessed against the national poverty line and indicative low disposable household income, both healthy and current (unhealthy) diets for Australian families living in major cities and inner regional areas were consistently unaffordable in all States and Territories. Our estimates in this national study align with our previous findings for eight areas with major supermarkets in Victoria in 2018, whereby healthy and current (unhealthy) diets were found to cost 33 % and 40 % of the national poverty line (income) and 26 % and 31 % of the indicative low disposable household income^(12)^. Additional evidence has suggested that diets are largely unaffordable in other parts of Australia where incomes are low, including in outer regional Victoria and remote communities^(13,15,37,38)^. Emerging international evidence is beginning to elaborate on our understanding of income-driven or *income-related food insecurity,* which is thought to affect one in ten people across sixteen European countries^(39)^.

### Household incomes in Australia

We further exemplified how income can drive differences in diet affordability by examining interstate differences according to median gross household income (the only available state-level metric). Our analyses suggest that healthy and current (unhealthy) diets were most *affordable* in major cities and inner regional areas of the NT and ACT where median gross household incomes are highest. Yet these results are likely to conceal income inequalities between professionally employed (e.g. mining) groups and other groups experiencing socio-economic disadvantage in the NT (inequalities that are also likely to exist in other jurisdictions). Of particular concern is how the median weekly gross household income for Aboriginal and Torres Strait Islander peoples in the NT ($1225/week)^(40)^ is 38 % less than the median weekly household income for the whole territory ($1983/week)^(41)^. When using the median gross household income for Aboriginal and Torres Strait Islanders in the NT, a healthy diet becomes overtly unaffordable (48 % of this income, aligning with previous research^(15)^). Literature increasingly points towards the importance of income and poverty as significant determinants of diet affordability – which in turn underscores the need to better consider food and beverage prices from a social systems perspective.

### Strengths and limitations

A key strength of the current study was the collection of data online and by phone calls, which reduced the financial expenditure and time required for food and beverage price collection, enabling simultaneous data collection across all Australian States and Territories for the first time. The reliability of these estimates can be inferred from a number of previous studies which have used the in-store HD-ASAP approach across a smaller number of areas^(12,42)^. For example, using data collected in-store in 2015, Lee *et al.* found that a healthy diet cost $603 and $627 in two major cities in New South Wales (compared with our state-wide estimate of $594 in 2019) and current diets cost $730 and $761 (compared with our state-wide estimate of $748)^(42)^.

Nonetheless, our sampling revealed that only major cities and inner regional areas contained both major supermarkets, which precluded diet pricing evaluations across three other ARIA^+^ classifications (outer regional, remote and very remote areas). The presence of major supermarkets was particularly limited in the NT (and completely absent in the Territory’s most disadvantaged IRSD quintiles) compared with most other States and Territories (see online supplemental Tables S3 and S4). Our price collection methods are further hindered in outer regional and remote areas by the absence of online food and beverage pricing data for the dominant independent grocers, including Independent Grocers of Australia.

### Implications for research and policy

Additional research is required to extend our low-resource data collection methods to parts of Australia (and the world) that do not have major online supermarkets, including remote communities where small stores are the main source of food and food prices have been repeatedly shown to be up to 60 % more expensive^(37,43,44)^. Citizen science or crowdsourcing approaches may be one useful way to engage community members in price collection using novel digital platforms^(45,46)^. Such approaches can also empower everyday citizens to be agents of change and contribute to the development of local food and economic policies that protect and promote diet-related health. In the meantime, our study strengthens the rationale for governments to continue funding in-store data collection of food and beverage prices in rural, remote and very remote settings.

Traditional food and beverage price monitoring methods, including the HD-ASAP approach, are also typically limited in their inclusion of diet variety and actual consumption/expenditure practices. Our methods have the potential to address these issues into the future; for example, by developing large food price data sets to examine the cost and affordability of different diet patterns, product types and prices, thereby leading to estimates that may better reflect the variability of population diets.

To inform more robust diet affordability calculations into the future, the available income data will need to be improved to include measures of median disposable household income (according to socio-economic position, remoteness and Aboriginal and Torres Strait Islander status). Comprehensive monitoring of food and beverage costs and their affordability can ultimately inform food pricing policy actions that can improve the healthiness of population purchases – especially among those experiencing socio-economic hardship^(37,43,47)^. This may include providing data to show exactly how more unaffordable healthy diets would become if the GST base was extended to include basic healthy foods or identifying specific food groups where pricing policies may be targeted (e.g. Sugar Sweetened Beverage taxes and regulations on supermarket price promotions)^(16)^. Social protection policies should also be revised to improve the social determinants of health (i.e. income, employment and housing) and the affordability of basic necessities in Australia^(38,39,48)^.

## Conclusions

Our study demonstrates that whilst the cost of a healthy and current (unhealthy) diet may be similar across major cities and inner regional areas (*n* fifty-one areas which have major supermarkets) in all eight Australian States and Territories, differences in diet affordability are apparent. Notably, a healthy diet remains unaffordable for families living below the poverty line, who are also at greater risk of diet-related ill-health^(49)^. To reduce inequities in diet-related disease and death in Australia, it is essential that food, social and economic policies are enacted to promote the economic appeal of healthy over current (unhealthy) diets and ensure that everyone receives a sufficient income which supports key opportunities to be healthy. This is particularly important now as the world experiences radical shifts in pricing and income structures due to unprecedented climate and health pandemics^(38)^. The development and application of robust food and beverage price monitoring systems (to inform effective policy actions) is also arguably more important now, than ever before.

## Supplementary material

For supplementary material accompanying this paper visit https://doi.org/10.1017/S1368980021004006. click here to view supplementary material
