# Supplementary material for: Costing recommended (healthy) and current (unhealthy) diets in urban and inner regional areas of Australia using remote price collection methods
Source: Public Health Nutr. 2021 Sep 21;25(3):528–37. doi: 10.1017/S1368980021004006 (PMC9991756; doi:10.1017/S1368980021004006)
Supplement: Supplementary file 1 [file S1368980021004006sup001.docx]

**SUPPLEMENTARY INFORMATION:**

Costing recommended (healthy) and current (unhealthy) diets in urban and inner regional areas of Australia using remote price collection methods

**Table S1: Mean fortnightly price ($AUD) of a healthy and unhealthy diet per amounts consumed by a reference household of two adults and two children, by IRSD***** **and ARIA^+§^ category and State and Territory, May 2019**

| **State/ Territory** | **ARIA** | **ARIA^+^ 1: Major cities** | | | | | **ARIA^+^ 2: Inner regional areas** | | | | |
| --- | --- | --- | --- | --- | --- | --- | --- | --- | --- | --- | --- |
|  | **IRSD** | **Q1** | **Q2** | **Q3** | **Q4** | **Q5** | **Q1** | **Q2** | **Q3** | **Q4** | **Q5** |
| **NSW**^¥^ | Healthy | 588.49 | 594.81 | 593.78 | 595.29 | 594.72 | 593.78 | 592.75 | 597.63 | 591.33 | n/a |
|  | Unhealthy | 743.55 | 750.14 | 743.33 | 751.73 | 746.40 | 746.43 | 759.94 | 748.43 | 751.27 | n/a |
| **Vic** | Healthy | 603.76 | 602.24 | 602.24 | 607.03 | 600.11 | 602.91 | 602.21 | 602.24 | 602.24 | 602.24 |
|  | Unhealthy | 754.23 | 749.92 | 754.76 | 751.07 | 751.78 | 736.90 | 747.15 | 737.64 | 747.39 | 750.69 |
| **Qld** | Healthy | 589.12 | 587.27 | 593.30 | 589.63 | 593.90 | 586.21 | 597.63 | 590.01 | 583.00 | n/a |
|  | Unhealthy | 737.06 | 747.16 | 743.63 | 739.32 | 746.66 | 738.17 | 782.42 | 762.97 | 764.59 | n/a |
| **SA** | Healthy | 597.07 | 594.69 | n/a | 598.16 | n/a | 588.72 | n/a | 586.62 | n/a | n/a |
|  | Unhealthy | 735.12 | 741.16 | n/a | 744.85 | n/a | 740.68 | n/a | 744.48 | n/a | n/a |
| **WA** | Healthy | 586.81 | 586.78 | 591.75 | 572.78 | 591.78 | 586.59 | 580.16 | 586.38 | 590.44 | n/a |
|  | Unhealthy | 746.26 | 730.11 | 751.53 | 729.98 | 741.01 | 747.67 | 731.39 | 767.50 | 755.49 | n/a |
| **Tas** | Healthy | n/a | n/a | n/a | n/a | n/a | 590.38 | 591.11 | 591.85 | 589.27 | 591.49 |
|  | Unhealthy | n/a | n/a | n/a | n/a | n/a | 726.24 | 725.42 | 732.55 | 750.96 | 721.64 |
| **NT** | Healthy | n/a | n/a | n/a | n/a | n/a | n/a | n/a | 597.16 | 591.23 | 597.04 |
|  | Unhealthy | n/a | n/a | n/a | n/a | n/a | n/a | n/a | 766.10 | 760.25 | 767.69 |
| **ACT** | Healthy | n/a | n/a | n/a | 594.81 | 596.59 | n/a | n/a | n/a | n/a | n/a |
|  | Unhealthy | n/a | n/a | n/a | 760.19 | 751.29 | n/a | n/a | n/a | n/a | n/a |

**IRSD: Index of Relative Socio-economic Disadvantaged; Q1: most disadvantaged, Q5: least disadvantaged (1)*

**^§^***ARIA***^+^***: Accessibility/Remoteness Index of Australia; ARIA^+^ 1: ‘Major cities’, ARIA^+^ 2: ‘Inner regional areas’. Note: no major supermarkets were identified in ARIA***^+^** *categories >2 (2)*

*^¥^NSW: New South Wales, Vic: Victoria, Qld: Queensland, SA: South Australia, WA: Western Australia, Tas: Tasmania, NT: Northern Territory, ACT: Australian Capital Territory*

**References:**

1. Census of Population and Housing: Socio-Economic Indexes for Areas (SEIFA), Australia, 2016. IRSD (updated 27/03/18, accessed 20/08/20). Available from: <https://www.abs.gov.au/ausstats/abs@.nsf/Lookup/by%20Subject/2033.0.55.001~2016~Main%20Features~IRSD~19>
2. Australian Statistical Geography Standard (ASGS): Volume 5 - Remoteness Structure, July 2016. Australian Bureau of Statistics. (updated 16/03/18, accessed 20/08/20). Available from: <https://www.abs.gov.au/ausstats/abs@.nsf/PrimaryMainFeatures/1270.0.55.005?OpenDocument>

**Table S2: Assumptions underlying calculation of indicative low disposable household income**

| **Household characteristics** | Two parents (adult male, adult female) with two children (14-year-old boy, 8-year-old girl) |
| --- | --- |
| **Paid employment, adult male** | Minimum wage of $18.93 per hour for 38 hour week (1) |
| **Paid employment, adult female** | Minimum wage of $18.93 per hour for 6 hour week (1) |
| **Family Tax Benefit Part A** | Fortnightly payment of $420.70, plus annual supplement of $751.90 per child (2) |
| **Family Tax Benefit Part B** | Fortnightly payment of $108.64, plus annual supplement of $365.00 per family (3) |
| **Energy Supplement** | Fortnightly payment of $11.90 (4) |
| **Rent assistance** | Fortnightly payment of $161.58 (5) |
| **Income tax paid** | Annual income tax of $2325.94 (6, 7) |

**References:**

1. Fair Work Commission. National Minimum Wage Order 2018. Fair Work Act 2009 - Annual wage review (updated 25 June 2018, accessed 3 Sept 2019). Available from: <https://www.fwc.gov.au/documents/awardsandorders/html/pr606629.htm>.

2. Australian Government. Family Assistance Guide. 3.6.1 FTB Part A - historical rates (updated 10 Aug 2020, accessed 3 Sept 2020) Available from: <https://guides.dss.gov.au/family-assistance-guide/3/6/1>.

3. Australian Government. Family Assistance Guide. 3.6.3 FTB Part B - historical rates (updated 10 Aug 2020, accessed 3 Sept 2020) Available from: <https://guides.dss.gov.au/family-assistance-guide/3/6/3>.

4. Australian Government Services Australia. Payment rates on Family Tax Benefit (FTB) (updated 24 June 2019, accessed 3 Sept 2020). Available from: <https://www.servicesaustralia.gov.au/individuals/services/centrelink/energy-supplement/how-much-you-can-get/payment-rates-family-tax-benefit-ftb>.

5. Australian Government. Family Assistance Guide. 3.6.3 FTB rent assistance - historical rates & thresholds (updated 10 Aug 2020, accessed 3 Sept 2020) Available from: <https://guides.dss.gov.au/family-assistance-guide/3/6/2>.

6. Australian Government. Australian Tax Office. Low and middle income earner tax offsets (updated 15 Jun 2020, accessed 3 Sept 2020). Available from: <https://www.ato.gov.au/individuals/income-and-deductions/offsets-and-rebates/low-and-middle-income-earners/>.

7. Australian Government. Australian Tax Office. Individual income tax rates (updated 01 Jul 2020, accessed 3 Sept 2020). Available from: <https://www.ato.gov.au/Rates/Individual-income-tax-rates/>.

**Table S3-a: Sampled and missing SA2s across IRSD* quintiles of socioeconomic disadvantage and Australian States and Territories**

|  | **NSW** | **Vic** | **Qld** | **SA** | **WA** | **ACT** | **Tas** | **NT** | ***Total, n*** |
| --- | --- | --- | --- | --- | --- | --- | --- | --- | --- |
| Q1, n | 2 | 2 | 2 | 2 | 2 | n/a | 1 | n/a | *11* |
| Q2, n | 2 | 2 | 2 | 1 | 2 | n/a | 1 | n/a | *10* |
| Q3, n | 2 | 2 | 2 | 1 | 2 | n/a | 1 | 1 | *11* |
| Q4, n | 2 | 2 | 2 | 1 | 2 | 1 | 1 | 1 | *12* |
| Q5, n | 1 | 2 | 1 | n/a | 1 | 1 | 1 | 1 | *8* |
| **State SA2s, n** | **9** | **10** | **9** | **5** | **9** | **2** | **5** | **3** | ***52*** |

**IRSD: Index of Relative Socio-economic Disadvantage; Q1: most disadvantaged, Q5: least disadvantaged (1)*

**Table S3-b: Sampled and missing SA2s across two ARIA^+§^ categories of remoteness and Australian States and Territories**

|  | **NSW** | **Vic** | **Qld** | **SA** | **WA** | **ACT** | **Tas** | **NT** | ***Total, n*** |
| --- | --- | --- | --- | --- | --- | --- | --- | --- | --- |
| Major cities, n | 5 | 5 | 5 | 3 | 5 | 2 | 1 | n/a | *26* |
| Inner regional areas, n | 4 | 5 | 4 | 2 | 4 | n/a | 4 | 3 | *26* |
| **State SA2s, n** | **9** | **10** | **9** | **5** | **9** | **2** | **5** | **3** | ***52*** |

**^§^***ARIA^+^: Accessibility/Remoteness Index of Australia; ARIA^+^ 1: ‘Major cities’, ARIA^+^ 2: ‘Inner regional areas.’ Note: no major supermarkets were identified in ARIA^+^ categories >2 (2)*

**References:**

1. Census of Population and Housing: Socio-Economic Indexes for Areas (SEIFA), Australia, 2016. IRSD (updated 27/03/18, accessed 20/08/20). Available from: <https://www.abs.gov.au/ausstats/abs@.nsf/Lookup/by%20Subject/2033.0.55.001~2016~Main%20Features~IRSD~19>
2. Australian Statistical Geography Standard (ASGS): Volume 5 - Remoteness Structure, July 2016. Australian Bureau of Statistics. (updated 16/03/18, accessed 20/08/20). Available from: <https://www.abs.gov.au/ausstats/abs@.nsf/PrimaryMainFeatures/1270.0.55.005?OpenDocument>
